# Inversion Efficiency Model Yields Improved Accuracy in MP2RAGE‐Based *T*_1_ Mapping in the Human Brain at 7.0T

**DOI:** 10.1002/nbm.70067

**Published:** 2025-06-02

**Authors:** Hampus Olsson, Jan Ole Opheim, Mads Andersen, Carl Herrman, Max Lutz, Sonia Waiczies, Thoralf Niendorf, Gunther Helms

**Affiliations:** ^1^ Max‐Delbrück‐Center for Molecular Medicine in the Helmholtz Association (MDC) Berlin Ultrahigh Field Facility (B.U.F.F.) Berlin Germany; ^2^ Philips Copenhagen Denmark; ^3^ Lund Bioimaging Center (LBIC) Lund University Lund Sweden; ^4^ Physikalisch‐Technische Bundesanstalt (PTB) Braunschweig and Berlin Germany; ^5^ Experimental and Clinical Research Center (ECRC), a Joint Cooperation Between the Charité Medical Faculty and the Max‐Delbrück‐Center for Molecular Medicine in the Helmholtz Association Berlin Germany; ^6^ MRI.TOOLS GmbH Berlin Germany; ^7^ Department of Neurophysics Max Planck Institute for Human Cognitive and Brain Sciences Leipzig Germany; ^8^ Department of Medical Radiation Physics, Clinical Sciences Lund Lund University Lund Sweden

**Keywords:** 7T, human brain, MP2RAGE, parametric mapping, *T*_1_ quantification

## Abstract

Estimation of the longitudinal relaxation time *T*_1_ from the MP2RAGE pulse sequence is based on a monoexponential signal evolution model. However, magnetization transfer (MT) caused by the inversion pulse induces a fast relaxation component, which appears as a reduction in the efficiency of the inversion. This may explain the underestimation of *T*_1_ derived from MP2RAGE. To address this systematic bias, an “apparent” inversion efficiency (*f*_inv_) was introduced, which comprises all mechanisms that affect the inversion in the monoexponential MP2RAGE signal model. The model was then extended by calibrating an empirical linear dependence of *f*_inv_ on *R*_1_ = 1/*T*_1_, resulting in increased accuracy of the estimated *T*_1_. The apparent inversion efficiency *f*_inv_ and the apparent *T*_1_* (yielding *T*_1_ by auxiliary *B*_1_^+^ mapping) were mapped at 7T in healthy adults using phase‐sensitive inversion recovery (IR) with four consecutive RAGE trains (PS‐MP4RAGE) in conjunction with adiabatic inversion using time‐resampled (TR)–FOCI and hyperbolic secant pulses. Upon validation by conventional IR‐EPI, PS‐MP4RAGE was used to calibrate the linear *f*_inv_ model for the human brain. These 3D *T*_1_ maps also served as a reference to assess the improvement of the MP2RAGE‐based *T*_1_ estimates. The apparent inversion efficiency *f*_inv_ was consistently smaller in white matter (WM) than in gray matter (~0.73 vs. ~0.84). The difference in WM *T*_1_ between MP2RAGE and the reference PS‐MP4RAGE technique was reduced by more than 200 ms when using the suggested *f*_inv_ model. MT effects after spin inversion in MP2RAGE can be accounted for by calibrating the apparent inversion efficiency *f*_inv_ without introducing additional parameters. The proposed empirical model retains the *B*_1_^+^ compensation inherent to MP2RAGE and facilitates accurate *T*_1_ quantification in brain tissue.

## Graphical abstract

Quantification of the longitudinal relaxation time using the MP2RAGE pulse sequence and a monoexponential forward signal model may be underestimated because of MT effects introduced by the radiofrequency inversion pulse. We address this bias by identifying an empirical relationship between the “apparent inversion efficiency” and the longitudinal relaxation rate.

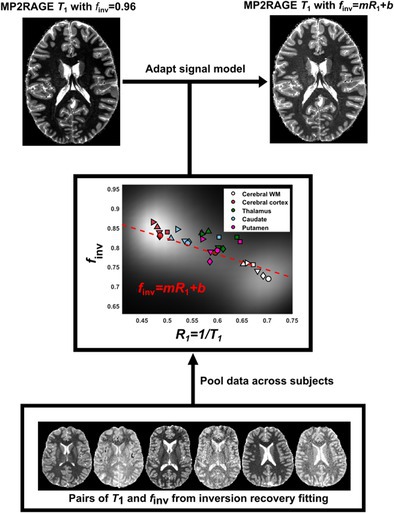

**Abbreviations**
*f*_inv_
: apparent inversion efficiency
PS‐MP4RAGE: Phase‐Sensitive Magnetization‐Prepared 4 Rapid Acquisition Gradient Echoes
HS: hyperbolic secant
MP2RAGE: Magnetization‐Prepared 2 Rapid Acquisition Gradient Echoes
TR‐FOCI: time‐resolved frequency offset corrected inversion

## 1 Introduction

The normalization of a *T*_1_‐weighted MPRAGE signal by a proton density (PD) weighted signal, acquired from two concatenated trains of rapid acquisition gradient echoes (RAGE), has been dubbed MP2RAGE [1]. MP2RAGE has become popular for high‐resolution structural MRI at 7T because it can compensate for inhomogeneities of the transmitted radiofrequency (RF) field (*B*_1_^+^). MP2RAGE allows for *T*_1_ mapping via a look‐up table of the modeled MP2RAGE intensities, thus permitting considerable variation in sequence timing. The longitudinal relaxation rate *R*_1_ = 1/*T*_1_ in brain parenchyma is used as a surrogate marker of myelination [2].

MP2RAGE tends to provide shorter *T*_1_ estimates than other techniques, especially in white matter (WM) [3, 4]. Such bias is likely due to an insufficient signal model used for *T*_1_ quantification. In MP2RAGE‐based *T*_1_ quantification, it is assumed that *T*_1_ is monoexponential and that the longitudinal magnetization, *M*_z_, is almost entirely inverted. Both approximations have been challenged owing to magnetization transfer (MT) effects induced by the inversion pulse [3] and potential *T*_2_ losses during adiabatic inversion [5, 6]. Although the latter will directly lead to reduced inversion efficiency, the equilibration of motion‐restricted protons and free water by a dynamic component faster than *T*_1_ will appear as an apparent reduction of the inversion efficiency in a monoexponential signal model [3]. The underlying spin‐physics dictates that the motion‐restricted pool cannot be inverted because of its very short *T*_2_, which means that some fraction of negative *M*_z_ will be transferred to the bound pool after inversion to re‐establish an equilibrium of exchange [7].

Aiming at an improved *T*_1_ quantification by MP2RAGE, we introduce an “apparent inversion efficiency” (*f*_inv_). This parameter comprises all mechanisms that affect the inversion in a hypothetically monoexponential *T*_1_‐driven signal evolution. For calibration and bias correction, we introduce a novel pulse sequence with concatenated identical RAGE trains to determine *f*_inv_ experimentally. The concatenated RAGE trains retain a monoexponential dynamic, thus avoiding extensive modeling of a multicompartment spin system that would require knowledge of additional parameters. Upon consistent observations of a strong correlation between *f*_inv_ and *T*_1_, we extended the forward signal model and introduced a *T*_1_‐dependent *f*_inv_ in the MP2RAGE‐based *T*_1_ calculation. We then calibrated *f*_inv_ for the time‐resampled (TR)–FOCI [8] and for a hyperbolic secant (HS) [9] inversion pulse, often used for adiabatic inversion. Here, it must be stressed that longitudinal relaxation is inherently biexponential, which may introduce bias to a two‐point model. The suggested approach does not model this issue with MP2RAGE‐based *T*_1_ mapping. We also do not consider partial volume effects because of spin environments with multiple *T*_1_. Rather, we suggest an empirically derived correction to mitigate observed differences between MP2RAGE and other *T*_1_ mapping techniques. We demonstrate that this simple amendment improves agreement between *T*_1_ maps obtained by inversion recovery (IR) with progressive small‐angle readout and by MP2RAGE without introducing additional parameters.

## 2 Methods

Experiments were performed on a 7T Philips Achieva system on software release 5.1.7.0 (Philips Healthcare, Best, NL) using a dual‐channel transmit head coil with a 32‐channel receive array (Nova Medical, Wilmington, MA). Healthy adult subjects volunteered after providing informed written consent as approved by the regional ethical review board.

### 2.1 MP2RAGE

Unless otherwise stated, the MP2RAGE protocol for structural MRI at 0.7‐mm isotropic resolution was employed as described in [10]. Here, a cycle duration (between two inversion pulses) of 5000 ms was achieved by reducing delays of free *T*_1_ relaxation. Acceleration by a factor of 2 using SENSE [11] and elliptical k‐space sampling by jittering adjacent k‐space increments [12] during the 256 excitations (turbo factor; TF) of the RAGE train resulted in a measurement time of 8:22 min for whole‐brain coverage. By choice of the RAGE train flip angles (*α*_1_/*α*_2_ = 5°/3°) and TI_1_/TI_2_ (900/2750 ms), a high contrast between WM, gray matter (GM), and cerebrospinal fluid (CSF) was prioritized over *B*_1_^+^ inhomogeneity compensation [10]. An “MT balanced” asymmetric sinc‐shaped RF pulse with a single side lobe and a fixed duration of 700 μs for both flip angles within TR = 6.8 ms were used for readout excitation [13].

Semi‐quantitative MP2RAGE images were calculated offline from real and imaginary valued signal components. *T*_1_ estimation was based on the MATLAB code provided with the original publication (URL: github.com/JosePMarques/MP2RAGE‐related‐scripts). An independently acquired auxiliary flip angle map was necessary to eliminate protocol‐dependent degrees of residual *B*_1_^+^ influence when determining *f*_inv_. Unless otherwise noted, rapid flip angle mapping with reduced bias was performed by combining three DREAM [14] maps as described previously [15].

### 2.2 Mapping *f*_inv_ and *T*_1_ With Phase‐Sensitive MP4RAGE (PS‐MP4RAGE)

3D maps of *f*_inv_ for a particular inversion pulse were obtained from the complex signals of four RAGE trains of low flip angle, applied in‐between inversions with minimal delays. The rationale behind this approach was to map *f*_inv_ and *T*_1_ similarly to a traditional IR model fitting approach while maintaining the speed and whole‐brain coverage of MPRAGE.

As in MP2RAGE, the signals of the four RAGE trains are acquired with consistent phase, reflecting the polarity of *M*_z_. When the last RAGE train represents positive *M*_z_, it can be used as a phase reference to wind back the phase of the previous RAGE signals. The signed signals (*S*_1,2,3,4_) of the real component then represent the polarity of *M*_z_ at each TI_1,2,3,4_ (at the readout of the zeroth k‐space line), hence “phase‐sensitive” (PS‐)MP4RAGE. Through pixelwise fitting of a monoexponential signal evolution, maps of *f*_inv_ are obtained along with the steady state signal, *S*_ss_, and the time constant with which the signal approaches *S*_ss_, *T*_1_*. The latter can be converted to *T*_1_ given the local flip angle [16, 17] to replace the *T*_1_ maps from a gold standard IR‐prepared approach.

The sequence parameters of PS‐MP4RAGE were as follows: Four sagittal volumes of FOV = 240 mm were acquired at 1.25 mm isotropic spatial resolution. Inversion times of TI_1,2,3,4_ = 725/2146/3576/5006 ms with a cycle duration of *t*_C_ = 5738 ms (with some variation between experiments to accommodate different inversion pulse durations). All 4 RAGEs used *α* = 2°, TR/TE = 7.45/2.94 ms, and TF = 192. As in the MP2RAGE implementation, asymmetric sinc pulses of 700 μs were used for readout excitation. Acceleration by elliptical k‐space coverage and SENSE factor 2 resulted in a 4:48‐min scan time.

This protocol was chosen so that *S*_2,3,4_ exhibits positive polarity for human brain tissue at 7T. Thus, a reference phase, $\varphi_{\mathrm{ref}}$, was determined as the mean phase of those signals. The signed signal of the first RAGE was then determined from the complex $S_{1}$ as the real component after correction by $\varphi_{\mathrm{ref}}$:

(1) $$S_{1,\text{signed}}=\left|S_{1}\right|\left(\cos\varphi_{1}\cos\varphi_{\mathrm{ref}}+\sin\varphi_{1}\sin\varphi_{\mathrm{ref}}\right).$$

A monoexponential transition was then fitted to the signed components $S\left({\mathrm{TI}}_{1,2,3,4}\right)$:

(2) $$S\left({\mathrm{TI}}_{1,2,3,4}\right)=S_{\mathrm{ss}}-\left(S_{\mathrm{ss}}-S_{\text{start}}\right)\exp\left(-\mathrm{TI}/T_{1}^{*}\right).$$

Here, $S_{\text{start}}$ is the signed signal immediately after inversion and $S_{\mathrm{ss}}$ is the steady state toward which the signal approaches with time constant $T_{1}^{*}<T_{1}$. From the signal at the end of the cycle $S_{\mathrm{end}}=S\left(t_{c}\right)$, the inversion efficiency is obtained as

(3) $$f_{\mathrm{inv}}=-S_{\text{start}}/S_{\mathrm{end}}.$$

The equation relating $T_{1}^{*}$ to $T_{1}$ [15] can be replaced by a rational approximation for small *α* << 1 rad and short TR << *T*_1_ [17]:

(4) $$T_{1}={\left(1/T_{1}^{*}-{\left(f_{T}\alpha \right)}^{2}/\left(2\mathrm{TR}\right)\right)}^{-1}$$

Here, $f_{T}$ is the ratio between the local and the nominal flip angle as obtained from auxiliary flip angle mapping.

*f*_inv_ mapping was performed on six healthy subjects for a TR‐FOCI pulse with 13‐μT peak *B*_1_ and 13‐ms duration [8]. On a subset of three subjects, an additional PS‐MP4RAGE was acquired using an HS pulse [9] of 15‐μT peak *B*_1_ and 21‐ms duration (corresponding to a nominal flip angle on the user interface of 1800°).

### 2.3 Calibrating *f*_inv_ in the MP2RAGE Forward Signal Model

The relation between *f*_inv_ and *R*_1_ = 1/*T*_1_ (see Section 3) was included in the forward signal model to address *f*_inv_‐related bias in MP2RAGE‐based *T*_1_ quantification. We used *R*_1_, rather than *T*_1_, because the former is more directly related to macromolecular content. Change in *f*_inv_ as a function of *R*_1_ was explored on a pixelwise level for the human brain. Pixels containing CSF were excluded from the analysis along with the globus pallidus, where *R*_1_ of the latter is strongly increased by iron content rather than MT. To remove outliers, pixels of the lowest and highest 5 percentiles were removed from both the *f*_inv_ and *R*_1_ estimates. The remaining pixels were pooled across subjects. A linear model was then fitted using the “robustfit” function of MATLAB R2021a (MathWorks Inc., Nantick, MA) for each inversion pulse.

The pulse‐specific linear *f*_inv_ models were implemented in the MP2RAGE‐based *T*_1_ calculation MATLAB package as provided with the original publication, rather than keeping the assumed *f*_inv_ constant. Thus, the value of *f*_inv_ is modified as the forward signal model loops through a vector of *T*_1_ values (50–5000 ms) when calculating the look‐up table. Of note, short *T*_1_ values are associated with a small *f*_inv_ and vice versa.

### 2.4 In Vivo Validation

The validation of the suggested MP2RAGE signal model was performed in two steps: (i) 2D validation of *T*_1_ and *f*_inv_ quantification obtained from PS‐MP4RAGE against single slice IR‐EPI and (ii) 3D validation of *T*_1_ quantification obtained from MP2RAGE against PS‐MP4RAGE. The generalizability of the approach was thereafter tested by (i) a multiprotocol validation on the same system and (ii) a multivendor validation using as similar protocols as possible.

#### 2.4.1 2D Validation of PS‐MP4RAGE Against IR‐EPI

Fully relaxed (TR = 30 s) single‐shot spin‐echo EPI with echo train length of 52, a bandwidth of 26.8 Hz/px in the phase‐encoding direction and a SENSE factor of 2.5 was acquired at 16 logarithmically spaced inversion times (TI = 200, 250, 310, 380, 470, 580, 720, 900, 1110, 1380, 1710, 2120, 2630, 3260, 4030, and 5000 ms). By choice of minimum TI = 200 ms, the fast component in the biexponential relaxation was omitted to allow for a monoexponential fit [18]. Inclusion of TI < 200 ms would require biexponential fitting and introduce instabilities in the fit. Thus, all TIs were chosen to allow monoexponential fitting. This validation step is confined to a single slice where the in‐plane resolution was 1.8 × 1.8 mm^2^ and the slice thickness was 5.4 mm. EPI distortions were partly corrected using a separately acquired *B*_0_ map and FSL FUGUE. For inversion, only the HS pulse was applied because the TR‐FOCI pulse was not readily available for the commercial IR‐EPI implementation.

Maps of *T*_1_ and *f*_inv_ obtained from PS‐MP4RAGE were compared with the corresponding IR‐EPI–derived maps for the brain of three subjects. The PS‐MP4RAGE maps were registered to the EPI slice and down‐sampled to the spatial resolution of the IR‐EPI maps using Free Surfer Freeview [19]. This approach allowed for direct visual inspection, as well as histogram‐based analysis.

#### 2.4.2 3D Validation of MP2RAGE Against PS‐MP4RAGE

**FIGURE 1:**
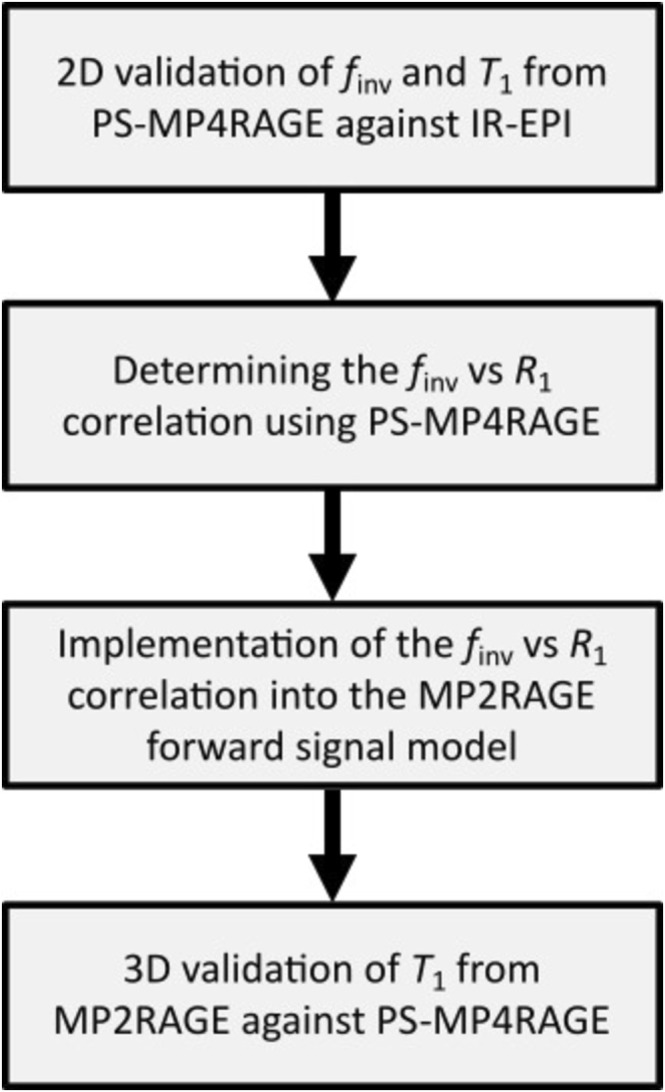
Flowchart depicting the high‐level experimental workflow.

Further to the validation of specific 2D slices, comparison with a 3D *T*_1_ mapping technique was performed to examine differences across the whole brain without any residual EPI distortions and fat displacements. For this reason, the *T*_1_ maps derived from PS‐MP4RAGE were compared with the MP2RAGE‐based *T*_1_ maps obtained from the suggested *f*_inv_ model in two healthy subjects. Both the HS and TR‐FOCI pulses were applied. MP2RAGE‐based *T*_1_ maps were computed using either a global *f*_inv_ = 0.96 as in the original work [1] or with our *T*_1_‐based *f*_inv_ model. The eight MP2RAGE‐based *T*_1_ maps (2 subjects × 2 inversion pulses × 2 *f*_inv_ settings) were compared with the four corresponding PS‐MP4RAGE‐based *T*_1_ maps (2 subjects × 2 inversion pulses) by calculating the percentage difference. In addition, 3D ROIs in 15 brain areas were evaluated to report some typical *T*_1_ estimates obtained with PS‐MP4RAGE and MP2RAGE with model‐based *f*_inv_. The mean *T*_1_ across the two subjects in each ROI was obtained for both mapping techniques and inversion pulses. The overall experimental workflow, from the 2D validation of PS‐MP4RAGE to the 3D validation of MP2RAGE, is outlined in the flowchart of Figure 1.

#### 2.4.3 Multiprotocol Validation

The reproducibility of derived *T*_1_ estimates across protocols with differing timings and flip angles was explored. One subject (male, 39 years old) was scanned with three separate MP2RAGE protocols with the following parameters:

- Protocol #1: Cycle duration = 5000 ms, TI_1_/TI_2_ = 900/2750 ms, *α*_1_/*α*_2_ = 5°/3°, TF = 256, TR = 6.8 ms, 0.70‐mm isotropic voxels. This was the default protocol used in this work and described in Section 2.1.
- Protocol #2: Cycle duration = 8250 ms, TI_1_/TI_2_ = 1000/3300 ms, *α*_1_/*α*_2_ = 7°/5°, TF = 160, TR = 6.9 ms, 1.0‐mm isotropic voxels. This was the originally suggested MP2RAGE protocol at 7T [1].
- Protocol #3: Cycle duration = 6000 ms, TI_1_/TI_2_ = 900/2700 ms, *α*_1_/*α*_2_ = 7°/5°, TF = 256, TR = 6.9 ms, 0.65‐mm isotropic voxels. Compared with Protocol #2, this is an accelerated variant prioritizing stronger WM‐GM contrast and higher spatial resolution at the cost of a stronger residual *B*_1_^+^ bias [20]. Note that TI_1_/TI_2_ was increased by 100 ms respectively compared with the reference to avoid having to use partial Fourier in the slice direction.

All protocols used the same TR‐FOCI pulse for inversion.

#### 2.4.4 Multivendor Validation

For translation of our approach to another MR platform, the *f*_inv_‐based signal model was tested on a 7T Magnetom MR system (Siemens Healthineers, Erlangen, Germany) using the same TR‐FOCI inversion for MP2RAGE (*n* = 1, 32 years old, male). Institutional Review Board Statement: The study was conducted in accordance with the Declaration of Helsinki and approved by the local ethics committee (Charité—University Medicine, Berlin, Germany, EA1/088/19).

Since the PS‐MP4RAGE sequence was not available at this system, validation was performed directly against the IR‐EPI protocol as described above but with an echo train length of 112 and a bandwidth in the phase‐encoding direction of 35.7 Hz/px. The MP2RAGE sequence was slightly altered to TR/TE = 7.30/2.55 ms, TF = 240 (“slices per 3D slab”): Parallel imaging (GRAPPA = 2) and partial Fourier in the phase‐encoding direction (pF = 6/8) were applied. The same TR‐FOCI pulse as described above was used. The *B*_1_^+^ map was obtained using actual flip angle imaging (AFI) [21] at 4‐mm isotropic resolution with TR_1_/TR_2_ = 20/120 ms, TE = 1.90 ms, *α* = 60°, GRAPPA = 2, sagittal FOV = 256 × 192 × 320 mm^3^ (AP × HF × RL) using both RF and gradient spoiling. MP2RAGE‐based *T*_1_ maps were calculated with both *f*_inv_ = 0.96 and the previously determined *f*_inv_ model for TR‐FOCI, registered to the axial slice of the distortion‐corrected EPI. The spatial resolution of MP2RAGE was down‐sampled to facilitate a comparison with the IR‐EPI‐based *T*_1_ map. The resulting three 2D *T*_1_ maps were compared using histogram analysis.

## 3 Results

**FIGURE 2:**
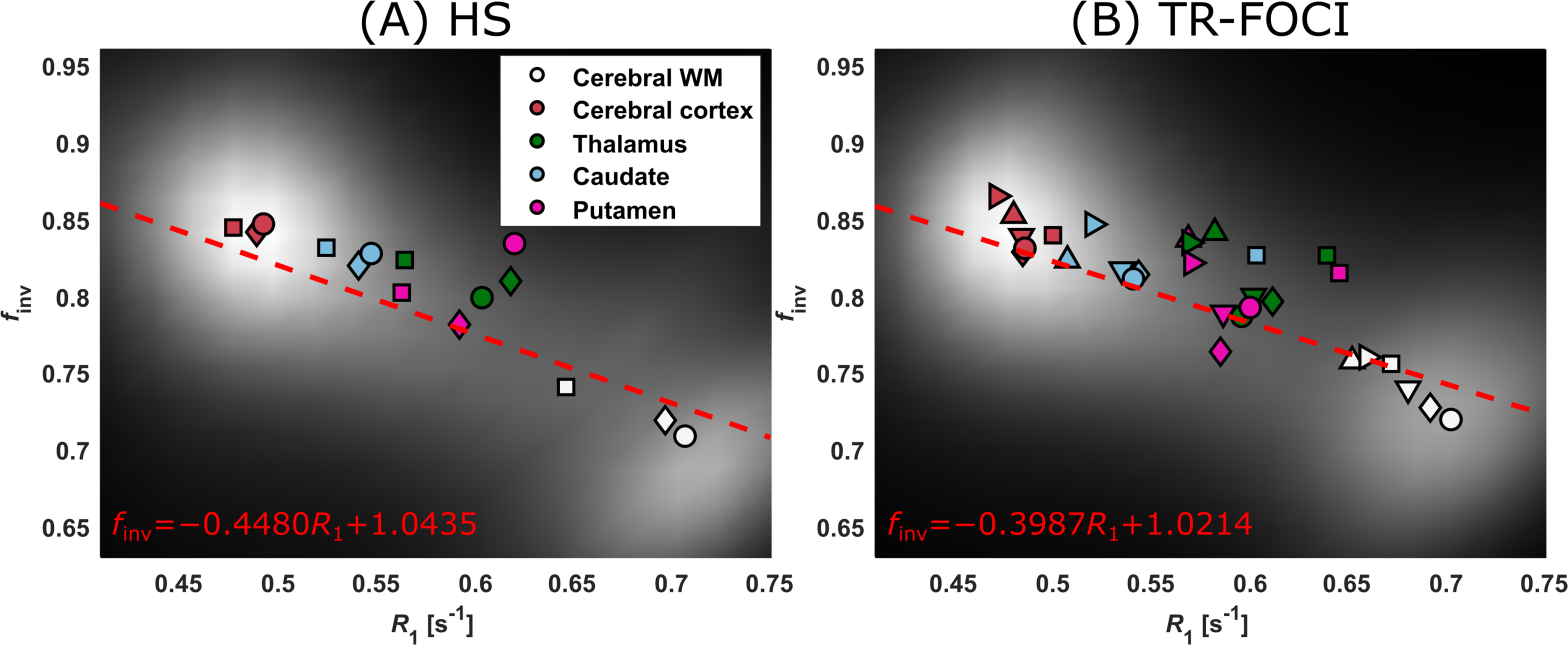
Inversion efficiency (*f*_inv_) as a function of *R*_1_. Plots show a linear fit (dashed red line and equation) of *f*_inv_ as a function of *R*_1_ when using (A) a hyperbolic secant pulse (HS, *B*_1,peak_ = 15 μT, *τ* = 21 ms) or (B) the TR‐FOCI pulse (*B*_1,peak_ = 13 μT, *τ* = 13 ms). The fits were based on brain pixels pooled across subjects and visualized as a density plot in grayscale showing two distinct aggregations of GM and WM. Colored markers show the median values of selected segmented brain regions (cerebral WM, cerebral cortex, thalamus, caudate, and putamen), where the shape of the markers denotes individual subjects (*n* = 3 in [A] and *n* = 6 in [B]). There is a clear negative correlation between *f*_inv_ and *R*_1_. Also note the flatter slope of the regression line obtained for the TR‐FOCI.

For all tested inversion pulses, mapping the inversion efficiency by PS‐MP4RAGE showed that *f*_inv_ in the human brain was smaller than 0.96 as assumed in the MP2RAGE forward signal model. The apparent inversion efficiency *f*_inv_ was consistently smaller in WM than in GM (~0.73 vs. ~0.84). Scatterplots of *f*_inv_ over *R*_1_ showed a cluster pattern representing GM and WM pixels (Figure 2) around *R*_1_ = 0.48 s^−1^ and *R*_1_ = 0.7 s^−1^, respectively. Inversion efficiency decreased with RF pulse duration and increased with RF pulse power indicating a combination of *T*_2_ losses during and MT effects after adiabatic inversion (see Figure S1 and S2).

### 3.1 Calibration of *f*_inv_ in the MP2RAGE Forward Signal Model

Figure 2 shows the two linear fits obtained for either HS or TR‐FOCI. Both show a negative correlation between *f*_inv_ and *R*_1_ with a steeper slope for the HS pulse (*f*_inv_ = −0.4480 s·*R*_1_ + 1.0435) than for the TR‐FOCI pulse (*f*_inv_ = −0.3987 s·*R*_1_ + 1.0214). For an assumed *R*_1_ = 0.25 s^−1^ of CSF, these lines extrapolate to *f*_inv_ = 0.923 (HS) and *f*_inv_ = 0.922 (TR‐FOCI). The GM and WM clusters are interpolated to half‐distance at *R*_1_ = 0.59 s^−1^ yielding *f*_inv_ = 0.779 (HS) and 0.786 (TR‐FOCI). Note that several subjects were pooled to average the potential influence of individual *B*_1_^+^ distributions.

### 3.2 In Vivo Validation

#### 3.2.1 2D Validation of PS‐MP4RAGE Against IR‐EPI

**FIGURE 3:**
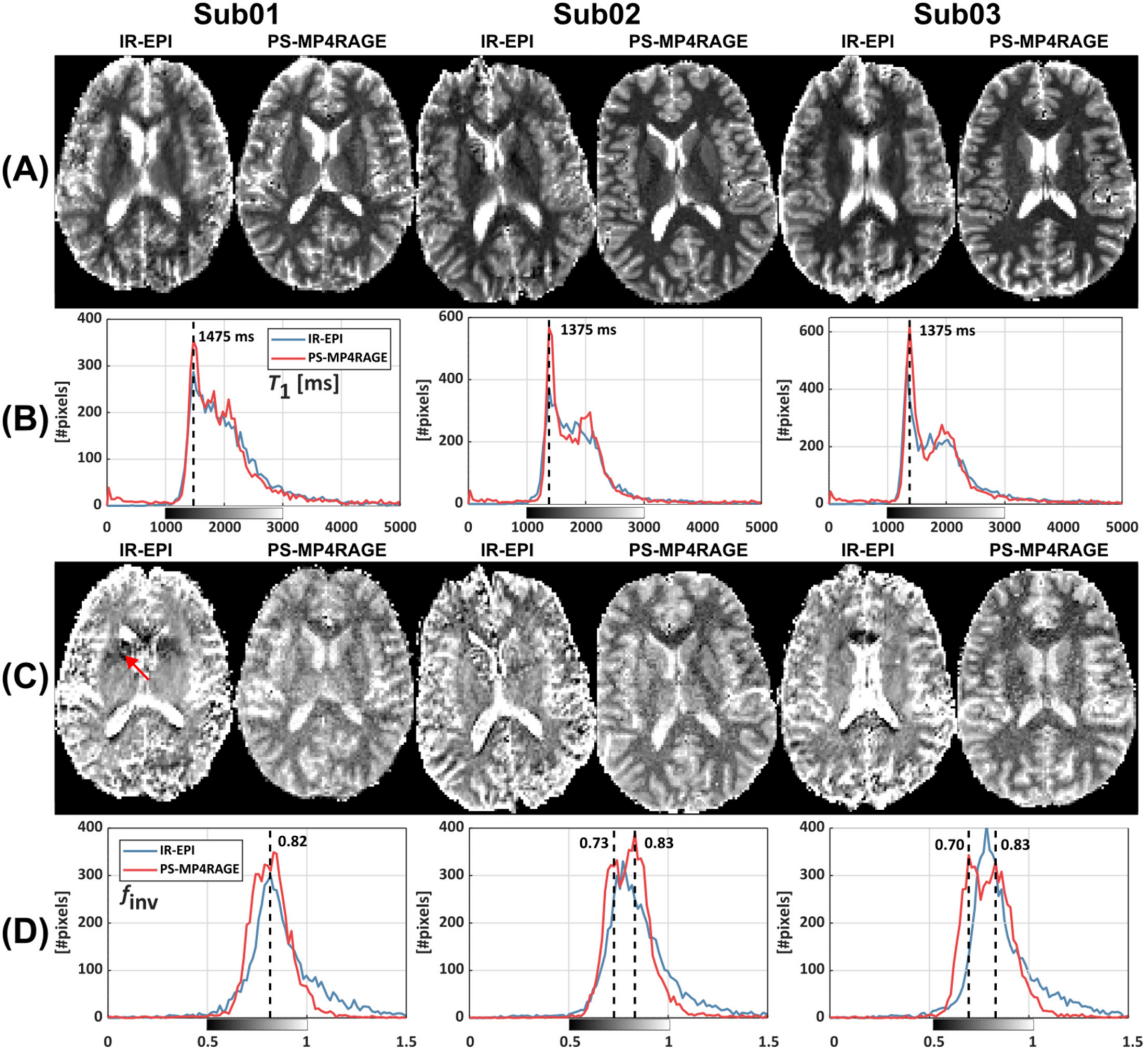
In vivo validation of PS‐MP4RAGE for *T*_1_ and *f*_inv_ maps. (A) Maps of *T*_1_ obtained from IR‐EPI or PS‐MP4RAGE in three healthy subjects. (B) Histograms of the *T*_1_ relaxation times for the same slice shown in (A). Note the common WM peaks (dashed lines). (C) Maps of *f*_inv_ obtained from IR‐EPI or PS‐MP4RAGE in three healthy subjects. Although artifacts and noise are more prevalent in the IR‐EPI maps, overall estimates are comparable. (D) *f*_inv_ histograms obtained from the *f*_inv_ maps in (C). For Sub02 and Sub03, distinct WM and GM peaks are distinguishable with PS‐MP4RAGE (dashed lines). Note the two peaks of GM and WM in the *f*_inv_ histograms derived from PS‐MP4RAGE. Red arrow denotes a chemical shift artifact.

PS‐MP4RAGE–derived *T*_1_ maps agreed well with those obtained from IR‐EPI in all subjects (Figure 3, rows A and B). Across the three subjects, ROI analysis in frontal WM, caudate head, and the frontal horn of the lateral ventricle (right hemisphere) resulted in averages of 1380 ± 63/1807 ± 91/4301 ± 101 ms for IR‐EPI and 1414 ± 49/1886 ± 38/4764 ± 225 ms for PS‐MP4RAGE.

The corresponding *f*_inv_ maps were in reasonable agreement for the brain parenchyma overall in view of the higher signal variations in IR‐EPI (Figure 3, rows C and D). However, the WM‐GM contrast was much less pronounced for IR‐EPI compared with PS‐MP4RAGE. IR‐EPI‐based *f*_inv_ estimates often exceeded 1 in CSF pixels, possibly indicating influence from slice profile effects. A corresponding ROI analysis as above (WM/GM/CSF) yielded 0.76 ± 0.03/0.76 ± 0.03/1.00 ± 0.05 for IR‐EPI and 0.69 ± 0.03/0.83 ± 0.01/0.97 ± 0.02 for PS‐MP4RAGE. The IR‐EPI‐based *f*_inv_ maps also showed artefacts due to fast‐relaxing residual fat signals (Figure 3, row C, red arrow). This underlines the usefulness of PS‐MP4RAGE for the dual purpose of calibrating the *f*_inv_ model and providing a 3D reference for *T*_1_ estimates. In Subjects 2 and 3, very low estimates of *f*_inv_ are observed in the genu of the corpus callosum, possibly indicative of the increased amount of nerve fibers present. However, this reduction compared with surrounding WM was not very well reproduced between IR‐EPI and PS‐MP4RAGE.

#### 3.2.2 3D Validation of MP2RAGE Against PS‐MP4RAGE

**FIGURE 4:**
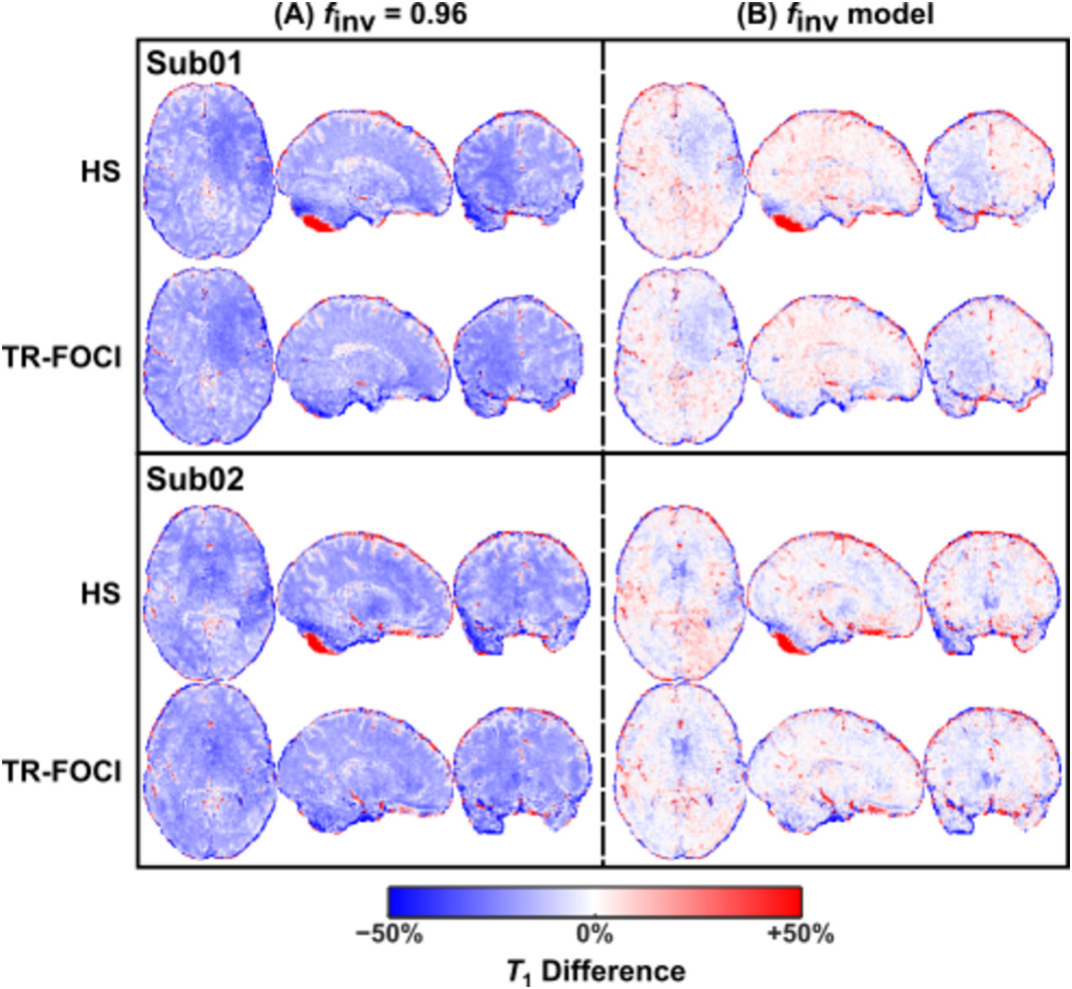
Difference maps showing the improved agreement between MP2RAGE‐based and PS‐MP4RAGE‐based *T*_1_ estimates when using the suggested *f*_inv_ model compared with a default global value of *f*_inv_ = 0.96. The underestimation observed using *f*_inv_ = 0.96 (left Column A) was strongly reduced when applying the *f*_inv_ model (right Column B) for both the HS (first and third rows) and TR‐FOCI (second and fourth rows) inversion pulse and was reproducible across the two subjects (upper and lower half of figure). Note also the failed adiabatic inversion using HS in the low *B*_1_^+^ region of the cerebellum visible in the sagittal slices.

**FIGURE 5:**
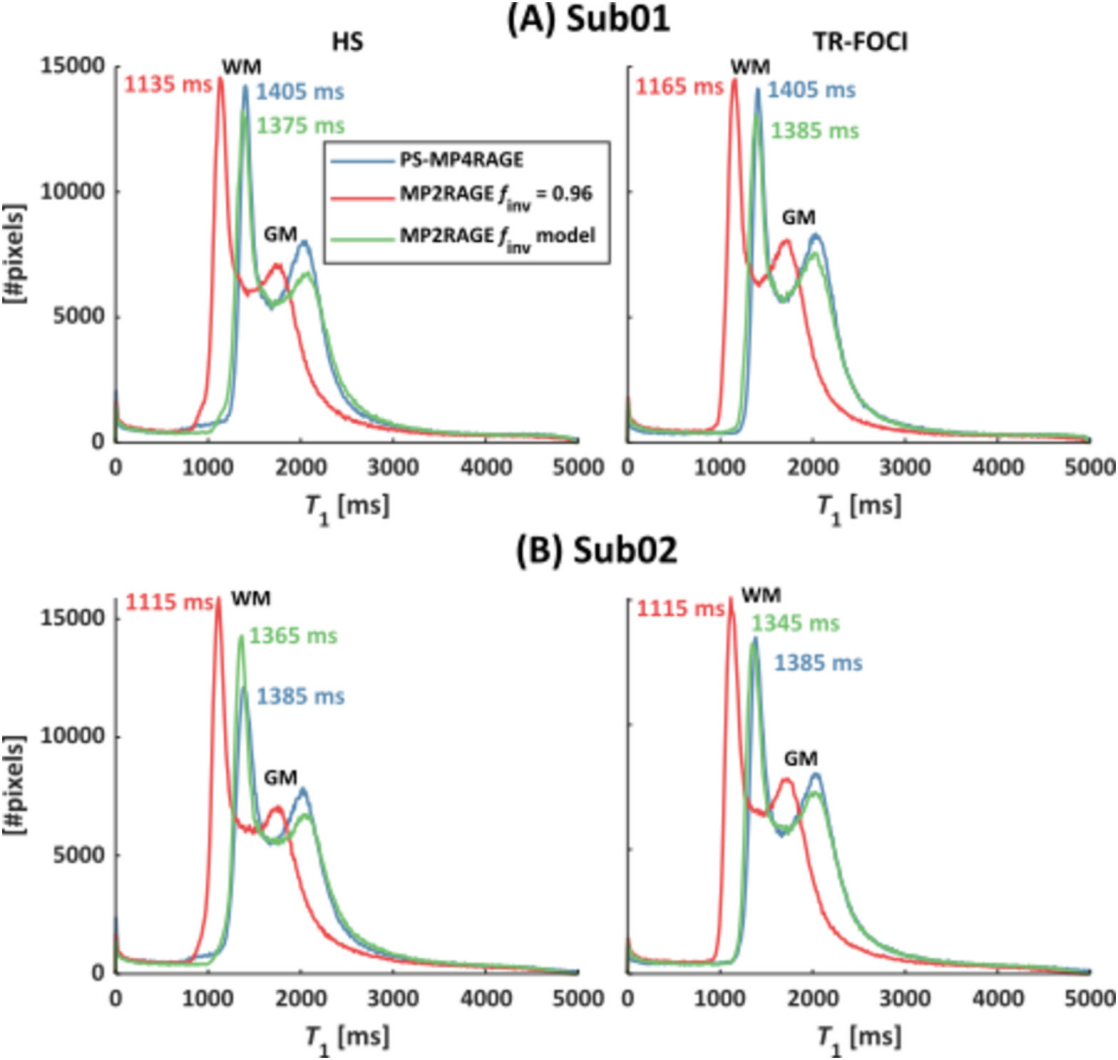
*T*_1_ histograms obtained for two subjects (A, B) and two inversion pulses (left/right column). The peak value of the mode representing WM reveals a difference of over 200 ms between MP2RAGE *f*_inv_ = 0.96 (red) compared with PS‐MP4RAGE (blue) and MP2RAGE with the linear *f*_inv_ model (green). This demonstrates that the underestimation in MP2RAGE‐based *T*_1_ compared with PS‐MP4RAGE is largely compensated when using the suggested *f*_inv_ model.

For validation, difference maps between the *T*_1_ maps obtained from MP2RAGE (with specific *f*_inv_ settings) and the corresponding PS‐MP4RAGE reference were calculated for each inversion pulse (Figure 4). The underestimation obtained with *f*_inv_ = 0.96 is drastically reduced (e.g., from ~−21% to ~−4% in frontal WM). The color scale highlights a spatial pattern of positive and negative bias, which varies with subject and between scans. This spatial pattern was not reproduced in the respective *B*_1_^+^ maps (Figure S3 and Table S4). The corresponding whole‐brain histograms (Figure 5) demonstrate the improvement of *T*_1_ quantification using the extended signal model. However, *T*_1_ in CSF was consistently shorter with the linear model. The residual deviation between PS‐MP4RAGE and MP2RAGE with model‐based *f*_inv_ was also assessed by ROI analysis in 15 areas (Table 1). The average deviation across all brain parenchyma ROIs (excluding CSF) was −1.8% for the HS pulse and −2.5% for the TR‐FOCI. Note that *T*_1_ relaxation times derived from using the HS and TR‐FOCI pulses were in good agreement. This result is in accordance with the *f*_inv_ over *R*_1_ = 1/*T*_1_ dependence shown in Figure 2. Averaged over both techniques, both pulses and all three subjects, we observed a *T*_1_ of 1334 ± 37 ms in WM (frontal, posterior, and corpus callosum) and 2163 ± 23 ms in cortical GM.

**TABLE 1:** *T*_1_ ROI analysis. The average *T*_1_ across the two subjects ± standard deviation. The relative difference between MP2RAGE using the *f*_inv_ model and PS‐MP4RAGE is reported for the HS and TR‐FOCI inversion pulse, respectively.

	HS PS‐MP4RAGE	HS MP2RAGE	*T*_1_ relative difference (%)	TR‐FOCI PS‐MP4RAGE	TR‐FOCI MP2RAGE	*T*_1_ relative difference (%)
Frontal WM (L)	1366 ± 14	1327 ± 29	−2.9	1370 ± 10	1329 ± 35	−3.0
Frontal WM (R)	1387 ± 14	1289 ± 10	−7.1	1367 ± 45	1288 ± 6	−5.8
Posterior WM (L)	1330 ± 40	1331 ± 65	+0.1	1350 ± 9	1332 ± 52	−1.3
Posterior WM (R)	1357 ± 68	1383 ± 28	+1.9	1352 ± 24	1363 ± 24	+0.8
Genu	1315 ± 73	1232 ± 40	−6.3	1291 ± 56	1273 ± 42	−1.4
Splenium	1352 ± 17	1337 ± 20	−1.1	1352 ± 30	1351 ± 11	−0.1
Putamen (L)	1701 ± 60	1698 ± 26	−0.2	1696 ± 73	1678 ± 42	−1.1
Putamen (R)	1670 ± 18	1629 ± 23	−2.5	1716 ± 10	1594 ± 25	−7.1
Caudate head (L)	1830 ± 4	1836 ± 12	+0.3	1820 ± 7	1821 ± 12	+0.1
Caudate head (R)	1865 ± 50	1732 ± 27	−7.1	1840 ± 41	1716 ± 28	−6.7
Thalamus (L)	1815 ± 47	1820 ± 28	+0.3	1847 ± 57	1805 ± 13	−2.3
Thalamus (R)	1817 ± 4	1833 ± 17	+0.9	1831 ± 9	1801 ± 11	−1.6
Cortical GM	2172 ± 70	2159 ± 83	−0.6	2193 ± 87	2129 ± 80	−2.9
Frontal horn (L)	4824 ± 251	4213 ± 47	−12.7	4803 ± 236	4294 ± 51	−10.6
Frontal horn (R)	4644 ± 353	3859 ± 65	−16.9	4693 ± 364	3860 ± 29	−17.7

#### 3.2.3 Multiprotocol Validation

**FIGURE 6:**
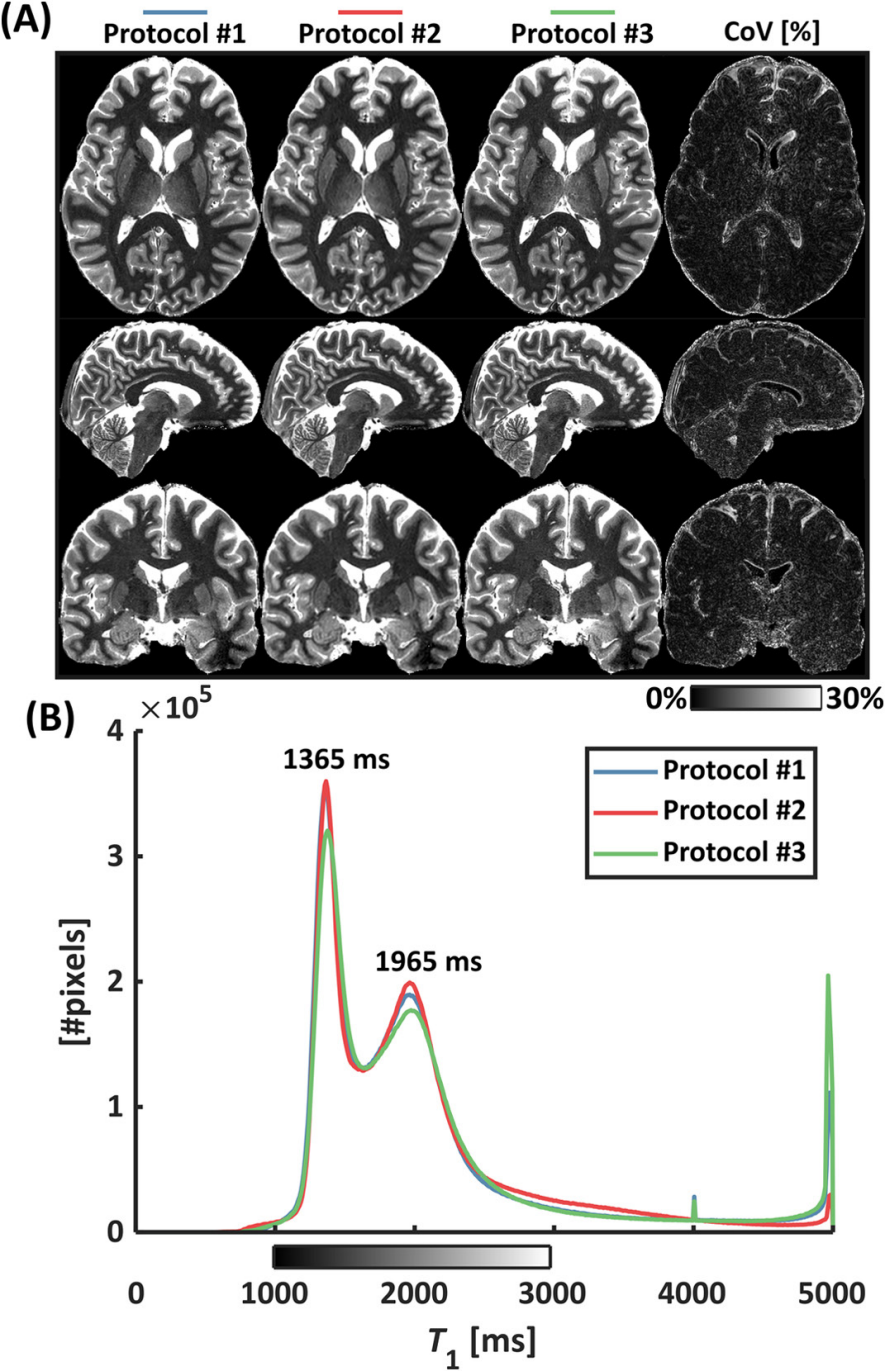
*T*_1_ maps (A) obtained with three separate protocols but with the same TR‐FOCI inversion pulse and *f*_inv_ model. The CoV (A, rightmost column) and whole‐brain histograms (B) reveal a good agreement between protocols with coinciding WM/GM modes at 1365/1965 ms, respectively.

MP2RAGE data from the three separate protocols were coregistered to the highest resolution and showed good agreement as assessed by the coefficient of variation (%) and histogram analysis (Figure 6). Segmentation of the whole‐brain WM and GM was performed with FSL FAST. Medians were 1391/1395/1411 ms in the WM and 2090/2101/2115 ms in the GM for Protocol #1, Protocol #2, and Protocol #3, respectively.

#### 3.2.4 Multivendor Validation

**FIGURE 7:**
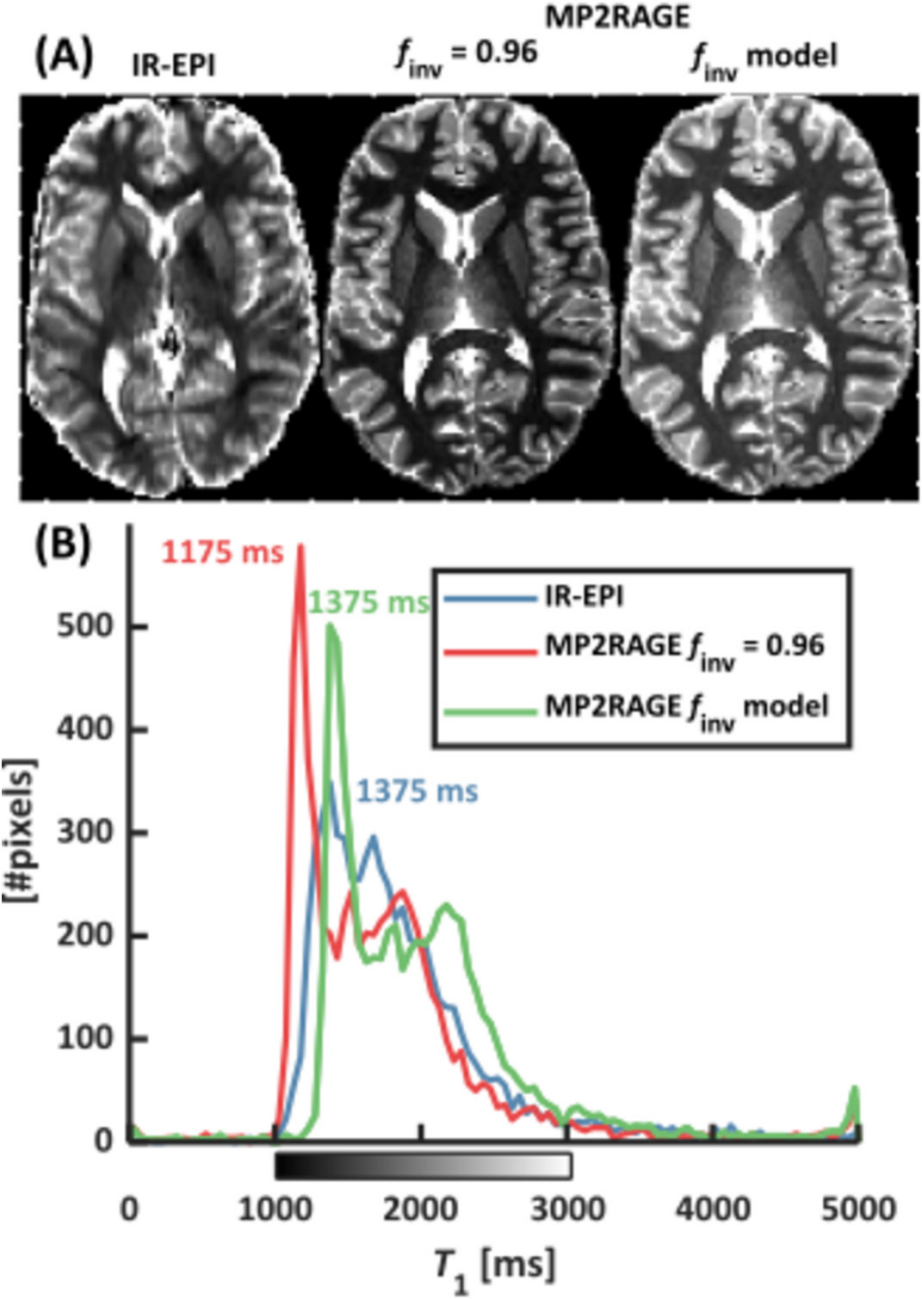
Axial IR‐EPI‐ and MP2RAGE‐based *T*_1_ maps (A) and corresponding histograms (B) acquired on a Siemens TRIO 7T system. The default setting of *f*_inv_ = 0.96 yielded an underestimation of *T*_1_. Here, however, the *f*_inv_ model calibrated on the Philips system introduced an overcompensation to the Siemens MP2RAGE *T*_1_ map.

Figure 7 shows the IR‐EPI‐based and MP2RAGE‐based *T*_1_ maps along with the corresponding histograms. Although the underestimation of relative IR‐EPI is reproduced for *f*_inv_ = 0.96 (~−8.0% in frontal WM), an overcompensation in frontal WM was observed with the *f*_inv_ model (~ + 11%). One possible explanation for this discrepancy between systems could be the different flip angle mapping techniques used for *B*_1_^+^ correction (DREAM vs. AFI).

## 4 Discussion

This work demonstrates the feasibility and value of an extension to the MP2RAGE signal model for improving the accuracy of *T*_1_ mapping in the human brain at the high spatial resolution and time efficiency of MP2RAGE. For calibration, apparent inversion efficiency (*f*_inv_) and *T*_1_ were mapped by a cycle of four consecutive RAGE trains at a low flip angle (PS‐MP4RAGE). In line with consecutive IR‐EPI measurements after equilibration by MT, this revealed longer *T*_1_ values than generally reported at 7T using IR, especially in WM [22, 23, 24].

We determined an empirical relation between *f*_inv_ and *R*_1_. This approach is free of introducing additional parameters and/or auxiliary maps into the MP2RAGE signal model. Specifically, our approach maintains MP2RAGE compensation for local *B*_1_^+^ in the dynamics of the free water signal. Correcting for incomplete inversion and MT yielded *R*_1_ values similar to those obtained with MT modeling [18]. We did not attempt to model the initial MT effects by extended Bloch equations, which are sensitive to the local *B*_1_^+^. Instead, the calibration was performed on pooled data to average out the influence of individual *B*_1_^+^ variations across subjects.

Note that *f*_inv_ represents an extrapolation to zero TI by monoexponential longitudinal relaxation as observed at sufficiently long TI to exclude the fast MT component (“apparent inversion efficiency”) and to ensure that free water and motion‐restricted protons are equilibrated. Use of signed signals in PS‐MP4RAGE simplifies the handling of residues (compared with magnitude IR) and may compensate for the limited number of signals acquired. The readout pulses approximately saturate both pools by similar degrees [13], so that no additional bias is introduced during the RAGE trains. The use of a 2° flip angle in the PS‐MP4RAGE experiments served the purpose of minimizing the differences between free relaxation and the dynamics driven by the RAGE train. Minor inconsistencies that may occur during the first 200 ms after inversion will influence the encoding of high spatial frequencies and have only a minor impact on the *T*_1_ contrast.

In supplementary measurements, the HS inversion pulse was systematically varied to isolate the mechanisms behind the reduced inversion efficiency. Here, the pulse duration was controlled via the (nominal) flip angle and peak *B*_1_ in the user interface. As previously identified by Bloch simulations for 9.4T, *f*_inv_ decreased with pulse duration due to *T*_2_ decay (Figure S1). The integrated RF power changes with the square of peak *B*_1_ at constant duration (Figure S2). As the bound pool is more saturated, less saturation is transferred from the inverted water, resulting in higher apparent inversion efficiency. Note that this effect could potentially also cause a spatial bias in *f*_inv_ due to the spatially varying transmit field at 7T. In other words, adiabatic inversion using pulses of rather long duration and low power is expected to exacerbate the reduction in *f*_inv_ and the associated underestimation of *T*_1_. The latter has been observed in exploratory experiments looking at variations of the MP2RAGE estimates of *T*_1_ [6]. In this previous work, local *f*_inv_ was estimated by comparison to target *T*_1_ values, as by Oran et al. [25]. Thus, the *f*_inv_ estimates will appear biased via the chosen target value for *T*_1_. Specifically, a shorter target *T*_1_ will shift the estimated *f*_inv_ to larger values, and vice versa. The effects of *T*_2_ and saturation transfer were both overcome by mapping *f*_inv_ via PS‐MP4RAGE. The spatial resolution, however, is lower than for MP2RAGE. Since PS‐MP4RAGE samples the approach to the driven equilibrium, higher resolution will prolong the RAGE trains and thus constrain the flip angle to even smaller values. This constitutes an additional constraint for balancing spatial resolution and SNR. In our PS‐MP4RAGE implementation, the number of RAGE trains was governed by the “FreeFactor” feature, jittering the phase encoding blips between adjacent values [12].

Any specific modeling of MT effects needs to account for local *B*_1_^+^ inhomogeneities using auxiliary *B*_1_^+^ maps. Such effects may also challenge the translation of our results to MP2RAGE data obtained on MR instruments provided by other vendors than those used in this work because the implementation of RF pulses may differ by vendor. *B*_1_^+^ mapping may improve reproducibility across sites, especially for the MP2RAGE protocol used here [9]. With the improved representation of *f*_inv_ in the signal model, *T*_1_ is shown to be longer than previously reported in the 7T literature [22, 23, 24].

To summarize, MT effects after spin inversion in MP2RAGE can be accounted for by calibrating the apparent inversion efficiency *f*_inv_ without introducing additional parameters. The proposed empirical model retains the *B*_1_^+^ compensation inherent to MP2RAGE and facilitates accurate *T*_1_ quantification in brain tissue.

## Conflicts of Interest

Jan Ole Opheim and Mads Andersen were employed by Philips when most of the experimental work was conducted. Hampus Olsson has since started employment at Philips.

## Supporting information


**Figure S1** Apparent inversion efficiency versus adiabatic inversion pulse duration. A clear negative correlation was observed between *f*_inv_ and pulse duration in the short (er) *T*_2_ brain parenchyma relative CSF.
Figure S2 Apparent *f*_inv_ versus adiabatic inversion peak *B*_1_. A trend of increasing *f*_inv_ with increasing peak *B*_1_ is observed with a plateau beyond 12 μT. Accordingly, regions of low *B*_1_^+^ exhibited lower *f*_inv_. The trend is stronger in the brain parenchyma compared to CSF although the difference is less accentuated than in the pulse duration experiment (Figure S1).
**Figure S3.** (A) Difference map with ROI in high‐difference area. (B) *B*_1_^+^ map with ROI in same location as (A). (C) Difference map with ROI in a low‐difference area. (D) *B*_1_^+^ map with ROI in same location as (C).
Table S4 Mean ± standard deviation of the ROIs in Figure S3.

## Data Availability

MATLAB scripts for MP2RAGE‐related processing are provided by the original authors (https://github.com/JosePMarques/MP2RAGE‐related‐scripts). The same script but with the R1‐dependent finv modification are available here: https://github.com/OlssonHampus/MP2RAGE_custom_finv. Image data is available upon reasonable request.
