# Supplementary material for: Inversion Efficiency Model Yields Improved Accuracy in MP2RAGE‐Based T 1 Mapping in the Human Brain at 7.0T
Source: NMR Biomed. 2025 Jun 2;38(7):e70067. doi: 10.1002/nbm.70067 (PMC12130670; doi:10.1002/nbm.70067)
Supplement: Supplementary file 1 — Figure S1 Apparent inversion efficiency versus adiabatic inversion pulse duration. A clear negative correlation was observed between f inv and pulse duration in the short (er) T 2 brain parenchyma relative CSF. Figure S2 Apparent f inv versus adiabatic inversion peak B 1. A trend of increasing f inv with increasing peak B 1 is observed with a plateau beyond 12 μT. Accordingly, regions of low B 1 + exhibited lower f inv. The trend is stronger in the brain parenchyma compared to CSF although the difference is less accentuated than in the pulse duration experiment (Figure S1). Figure S3. (A) Difference map with ROI in high‐difference area. (B) B 1 + map with ROI in same location as (A). (C) Difference map with ROI in a low‐difference area. (D) B 1 + map with ROI in same location as (C). Table S4 Mean ± standard deviation of the ROIs in Figure S3. [file NBM-38-e70067-s001.docx]

# Supplementary Material

## Adiabatic pulse duration and peak *B*_1_

To disentangle the reduction in *f*_inv_ stemming from *T*_2_ decay and the reduction stemming from MT, two additional PS-MP4RAGE experiments were performed: (i) To study the effect of *T*_2_, the duration of the HS pulse was changed through 15, 19, 24, 31, 43, and 61 ms while the RF power integral was kept constant by concomitantly decreasing peak *B*_1_ through 20, 18, 16, 14, 12, and 10 µT. (ii) To study the effect of MT, the power integral of the HS pulse was changed by varying peak *B*_1_ through 8, 10, 12, 14, 16, and 18 µT while keeping the duration of the pulse constant at 20.6 ms.

Increasing the pulse duration at constant pulse power revealed a trend of decreased *f*_inv_, by ~0.4±0.2 1/s in ROIs of WM and GM (Supplementary Figure 1). This is likely caused by *T*_2_-relaxation losses during adiabatic inversion.


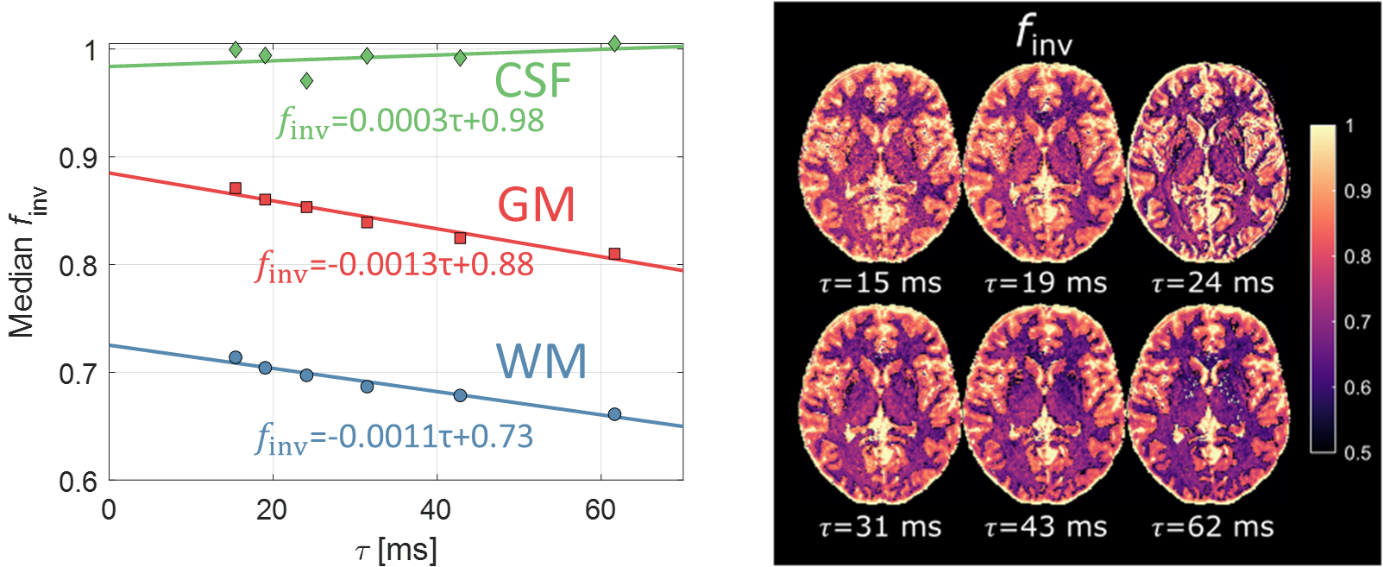


Supplementary Figure S1. Apparent inversion efficiency vs adiabatic inversion pulse duration. A clear negative correlation was observed between f_inv_ and pulse duration in the short(er) T_2_ brain parenchyma relative CSF.

A trend of increasing apparent *f*_inv_ was observed when increasing the adiabatic inversion peak *B*_1_ (Supplementary Figure S2). The trend was somewhat stronger in the brain parenchyma than in the CSF (which is to be expected) although results were much less conclusive than in the analogous pulse duration experiment (Supplementary Figure S1). *f*_inv_ approached an upper limit when the power of the inversion pulse was increased, as the bound pool was driven further into saturation. This plateau in *f*_inv_ appeared somewhere between 12 and 15 µT. It is expected that the dependence of *f*_inv_ on peak *B*_1_ observed here should be translatable also to the spatial variation of the transmit field at 7T. This aspect could be added to enhance the suggested model but the amount of data available for this work was not sufficient to implement this with any accuracy.


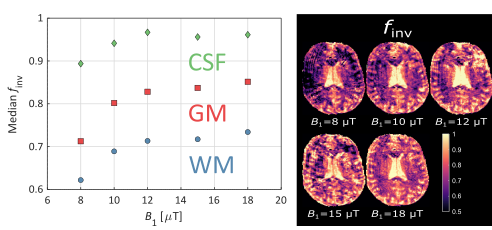


Supplementary Figure S2. Apparent f_inv_ vs adiabatic inversion peak B_1_. A trend of increasing f_inv_ with increasing peak B_1_ is observed with a plateau beyond 12 µT. Accordingly, regions of low B_1_^+^ exhibited lower f_inv_. The trend is stronger in the brain parenchyma compared to CSF although the difference is less accentuated than in the pulse duration experiment (Supplementary Figure S1).

## Residual differences in the 3D validation of MP2RAGE against PS-MP4RAGE

Some residual spatial differences in the *T*_1_ estimate of MP2RAGE and PS-MP4RAGE were observed (Figure 4). This difference was most notable in the anterior part of the brain between the right and left hemisphere of Sub01 (Figure 4, top row). To elucidate whether this difference was correlated to the transmit field, the difference map was compared to a co-registered *B*_1_^+^ map via ROI analysis (Supplementary Figure S3 and Supplementary Table S4). This comparison revealed no evident relationship between low/high *B*_1_^+^ and a large difference between MP2RAGE and PS-MP4RAGE *T*_1_ estimation. We speculate that the right-left difference could stem from slight variations between the transmit channel phase combination of the MP2RAGE and PS-MP4RAGE sequences respectively.


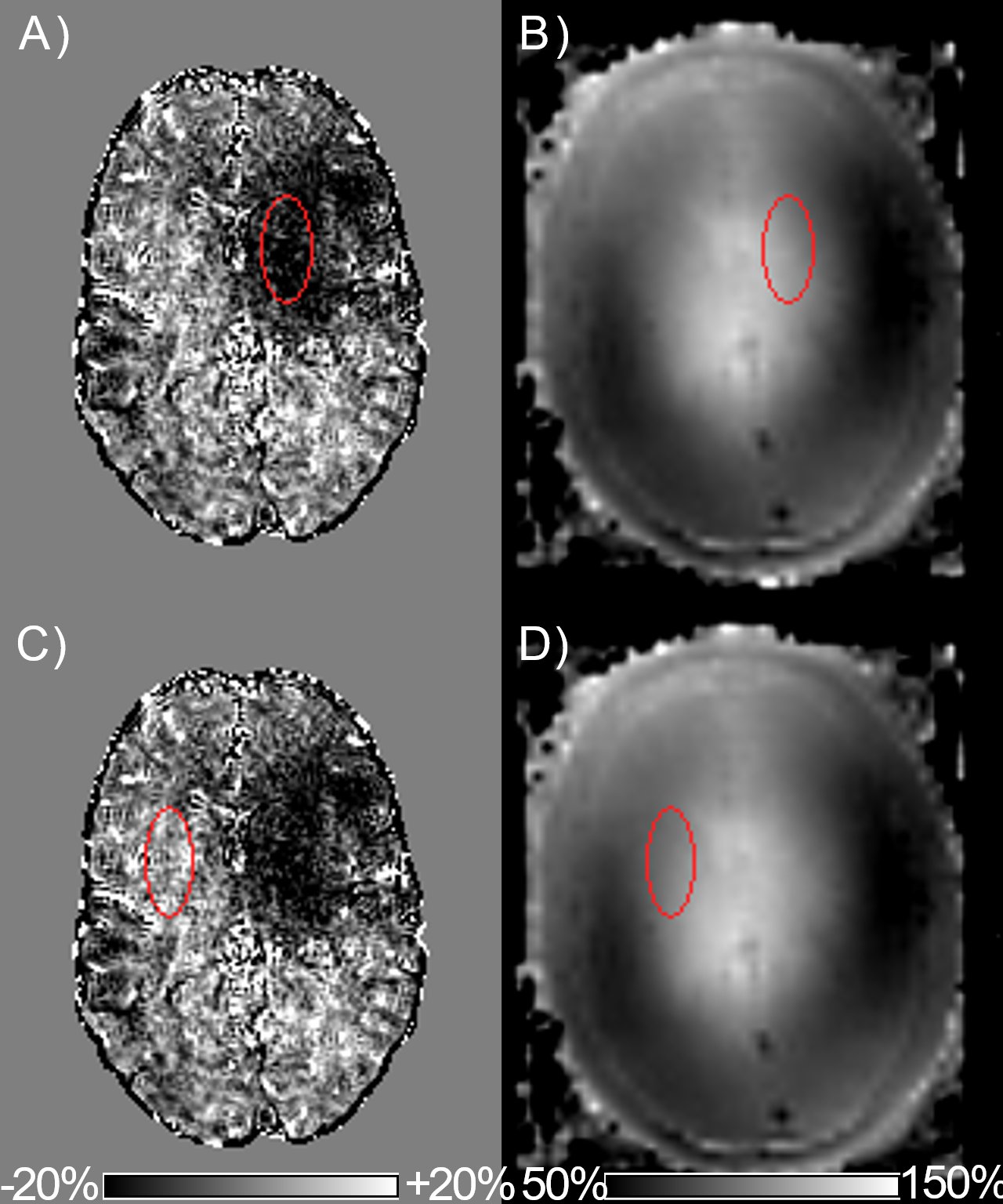


Supplementary Figure S3. A) Difference map with ROI in high-difference area. B) B_1_^+^ map with ROI in same location as A). C) Difference map with ROI in a low-difference area. D) B1+ map with ROI in same location as C).

Supplementary Table S4. Mean±standard deviation of the ROIs in Supplementary Figure S3.

| **Panel** | **Map** | **Mean±SD** |
| --- | --- | --- |
| A) | Difference | -9.5±2.8% |
| B) | B1+ | 106±10% |
| C) | Difference | +2.9±5.1% |
| D) | B1+ | 98±7% |
